# DCs at the center of help: Origins and evolution of the three-cell-type hypothesis

**DOI:** 10.1084/jem.20211519

**Published:** 2022-05-11

**Authors:** Renee Wu, Kenneth M. Murphy

**Affiliations:** 1 Department of Pathology and Immunology, School of Medicine, Washington University in St. Louis, St. Louis, MO

## Abstract

Last year was the 10th anniversary of Ralph Steinman’s Nobel Prize awarded for his discovery of dendritic cells (DCs), while next year brings the 50th anniversary of that discovery. Current models of anti-viral and anti-tumor immunity rest solidly on Steinman’s discovery of DCs, but also rely on two seemingly unrelated phenomena, also reported in the mid-1970s: the discoveries of “help” for cytolytic T cell responses by Cantor and Boyse in 1974 and “cross-priming” by Bevan in 1976. Decades of subsequent work, controversy, and conceptual changes have gradually merged these three discoveries into current models of cell-mediated immunity against viruses and tumors.

## Teaser

This review traces the separate discoveries of dendritic cells, cross-priming, and help for cytotoxic T cell responses. The authors document the gradual convergence of these discoveries into the current models of cell-mediated immunity, spotlighting unanswered questions and future directions.

## Introduction

Current models of anti-viral or anti-tumor immunity incorporate many interactions between various types of cells and many participating surface or signaling molecules and downstream pathways. The main effector cells are CD8 T cells recognizing antigens specific to viruses or tumors. These cells are primed by type I classical dendritic cells (cDC1s) that capture material from virally infected cells or tumor cells and present their antigens on MHC class I (MHC-I) molecules. These DCs can be “licensed” for effective priming by CD4 T cells that recognize antigen on MHC class II (MHC-II) molecules presented by the same cDC1. Recent work has confirmed or slightly advanced many details of this model, but it may be surprising that the essential features of these models were discovered nearly 50 yr ago.

**Figure 1.**
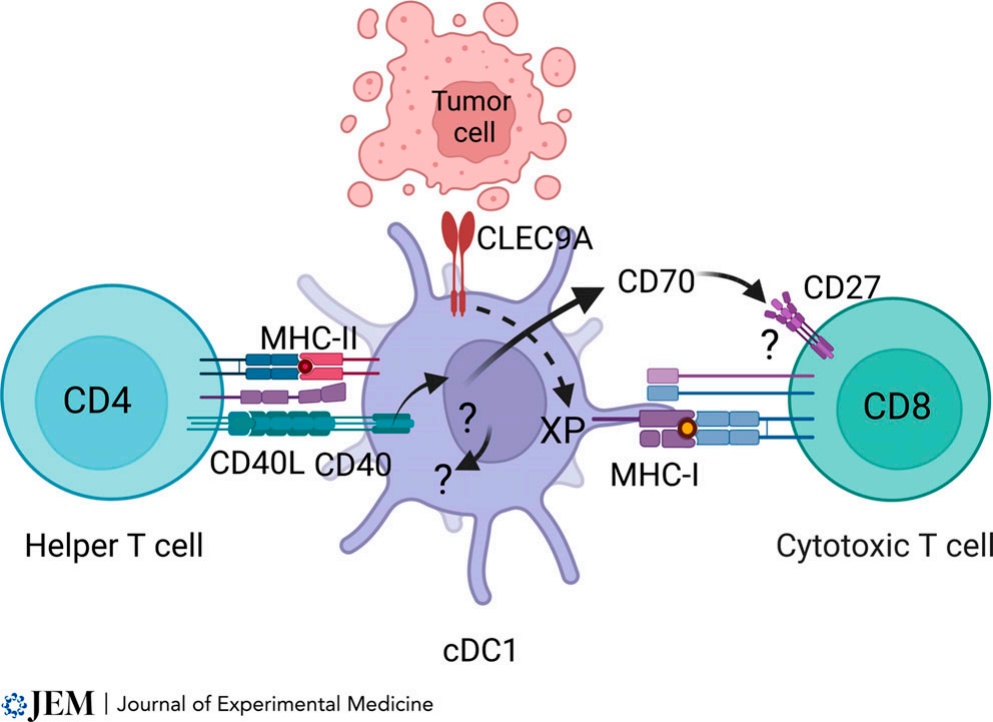
**A developing scheme for CD4 T cell–mediated help for CTL responses.** The cDC1 subset of cDCs can serve as an autonomous platform for priming both CD4 and CD8 T cells. The cDC1 captures and process cell-associated antigens for presentation by MHC-II molecules and cross-presentation (XP) for MHC-I molecules. CD4 T cell engagement induces surface expression of its CD40 ligand, stimulating CD40 signaling in cDC1 cells. This signaling enhances priming of CD8 T cells through mechanisms that remain incompletely defined, including induction of CD70 and potentially other costimulatory ligands, as well as DC-intrinsic effects.

There are three essential features in today’s current model (Laidlaw et al., 2016; Borst et al., 2018; Murphy and Murphy, 2022; Fig. 1). First, T cell priming is performed by a specialized APC belonging to DC lineages, which is separate from other types of APCs such macrophages, monocyte-derived DCs, or B cells. Second, CD8 T cell priming requires a specialized pathway of antigen processing in which exogenous antigens are captured and processed for loading MHC-I molecules, known as cross-presentation. Third, CD4 T cells “help” the priming of CD8 T cells by stimulating CD40 signaling in cDC1s that present cognate antigens on MHC-II molecules.

It is of some historical interest that these ideas all originated from independent discoveries made between 1973 and 1976, all published in *JEM*. In 1973, Steinman and Cohn reported the discovery of a new immune lineage they called “dendritic cells” (Steinman and Cohn, 1973). This discovery led to DCs eventually being recognized not only as distinct from macrophages, but also as comprising diverse subsets with distinct immune functions. In 1975, Cantor and Boyse reported the cooperation between different types of T cells in generating cytolytic T cell responses (Cantor and Boyse, 1975a). A long thread of subsequent studies on “help” led to today’s current appreciation of “DC licensing” via CD40 signaling that enhances CD8 T cell responses. In 1976, Bevan reported the phenomenon of in vivo “cross-priming” (Bevan, 1976) at a time when the nature of T cell recognition of antigen was poorly understood. As the field of antigen processing developed, the idea of processing exogenously derived antigens for presentation on MHC-I molecules remained controversial for decades, but is now firmly established as essential for many anti-viral responses and most anti-tumor immunity.

From today’s technically advanced viewpoint, it may be difficult to appreciate the importance of these original observations. Steinman’s original report of DCs is mostly descriptive, lacking the kind of functional data that is required for most reports today (Steinman and Cohn, 1973). The discovery of help for cytolytic T cell (CTL) responses by Cantor and Boyse uses archaic terminology, hindering ready accessibility (Cantor and Boyse, 1975a). Bevan’s cross-priming discovery relies on mouse genetics, somewhat unfamiliar to today’s students compared with more recent techniques (Bevan, 1976). These studies were published a decade before there was a solid understanding of how T cells recognize antigens (Shimonkevitz et al., 1984; Townsend et al., 1985). Moreover, the discovery of help for CTLs and cross-priming relied heavily on congenic mouse strains that allowed attribution of responses to specific elements of the H-2 locus (Snell, 1958; Snell and Jackson, 1958). As we will see, the first demonstration of cross-priming required use of specific congenic lines B10 and B10.D2 (Snell and Jackson, 1958), as well as BALB/c and BALB.B congenic strains (Freedman and Lilly, 1975). As such, these discoveries relied extensively on previous work by George Snell and others on tumor transplantation that generated these critical reagents. Here, we review the origins of these discoveries and trace their development and gradual fusion to form current models of cell-mediated immunity.

## Origins of DCs as drivers of adaptive immunity

### DCs as a distinct lineage

The discovery of DCs as a distinct type of immune cell originates from a paper published in the *JEM* in 1973 (Steinman and Cohn, 1973). This report was a morphological description of DCs without functional evidence of their importance, unlike current expectations of complete “stories” (Snyder, 2013). But this was only the first of a long series of studies by Steinman and colleagues, building a picture of DCs as a distinct immune lineage having critical and unique functions in adaptive immunity (Steinman et al., 1974; Steinman and Cohn, 1974; Steinman et al., 1975; Steinman et al., 1979; Nussenzweig and Steinman, 1980; Steinman et al., 1980), for which Steinman was awarded the 2011 Nobel Prize.

The discovery of DCs may have been partially motivated by earlier work that suggested the idea of “persistence of immunogenicity” in macrophages reported by Unanue in *JEM* (Unanue and Askonas, 1968) and elsewhere (Unanue, 1969; Unanue et al., 1969). Unanue’s studies originated the concept of antigen processing and stimulated interest in the fate of proteinaceous antigens following immunization. In the year before their discovery of DCs, Steinman and Cohn carried out work in an attempt to follow up this proposal by examining the fate of proteins such as horseradish peroxidase after phagocytosis by macrophages (Steinman and Cohn, 1972a; Steinman and Cohn, 1972b). Although their results were “difficult to equate” with the “persistence of protein antigens” (Steinman and Cohn, 1972b), they nonetheless may have motivated their examination of cells capable of capturing and processing antigens.

Subsequent work showed that DCs were distinct from other known lymphocytes or phagocytes (Steinman and Cohn, 1974), uncovered their rapid turnover and bone marrow origin (Steinman et al., 1974), identified their presence in mouse spleen (Steinman et al., 1975), showed their potency in primary mixed lymphocyte reactions (Steinman and Witmer, 1978), documented their high expression of MHC-II molecules (Steinman et al., 1979), and demonstrated their activity in the syngeneic mixed leukocyte reaction (Nussenzweig and Steinman, 1980). However, like the “persistence of immunogenicity,” the importance of DCs was also not appreciated universally at first. As recounted by William Paul, the early use of the mixed lymphocyte reaction to show DC’s capacity to expand T cells (Steinman and Witmer, 1978) led to uncertainty among some contemporary immunologists as to “the proper interpretation of the mixed leukocyte reaction data” (Paul, 2011). However, several years later, DCs were shown to be powerful APCs for T cells as well (Nussenzweig et al., 1980). Nonetheless, similarities between DCs and other myeloid lineages continued to be the basis for lingering reluctance in accepting DCs as a distinct lineage devoted to T cell priming (Hume, 2008).

### DCs as a heterogeneous group of cells

By the 1990s, DCs were recognized to comprise subtypes distinguished by distinct surface markers (Suss and Shortman, 1996; Kronin et al., 1996; Vremec et al., 1992; Wu et al., 1996; Shortman et al., 1995). The field today distinguishes cDCs studied by Steinman from plasmacytoid DCs (pDCs; Cella et al., 1999; Siegal et al., 1999). At least in murine models, it seems well established that only cDCs directly participate in presentation of antigens to T cells, while pDCs modify responses by secreting cytokines in response to the detection of viral infection (Swiecki and Colonna, 2015). The first monoclonal antibody to selectively identify cDCs, 33D1 (Nussenzweig and Steinman, 1982), was soon joined by NLDC-145, which recognizes DEC-205 (Jiang et al., 1995). CD8α^+^ was found to mark a subset of thymic-derived DCs (Shortman et al., 1995), and splenic cDCs subsets were distinguished on the basis of non-overlapping patterns of CD4 and CD8 expression (Shortman and Liu, 2002). Current surface markers used to distinguish murine cDCs include CD24, XCR1, CD172, and CD103 (Naik et al., 2007).

More recent work on DC development and function has been covered in several reviews (Liu and Nussenzweig, 2010; Merad et al., 2013; Murphy et al., 2016; Shortman and Heath, 2010; Durai and Murphy, 2016; Yin et al., 2021). Some notable findings include the demonstration that distinct DC subsets exhibit inherently different efficiencies for processing antigens for presentation by MHC-I and MHC-II molecules (Dudziak et al., 2007; Lehmann et al., 2017). cDC1s are preferentially specialized for MHC-I antigen processing and cDC2s for MHC-II antigen processing. However, the form of antigen, such as whether it is delivered as a soluble protein or in a cell-associated form, can also influence the efficiency of antigen presentation by DCs. Thus, studies have shown that cDC1 can process and present cell-associated antigens by MHC-II molecules (Kamphorst et al., 2010; Iyoda et al., 2002). This was confirmed using genetic systems that allow for the elimination of cDC1 in vivo (Hildner et al., 2008; Durai et al., 2019) or selective gene inactivation in cDC1 (Ferris et al., 2020). Currently, there is much activity aimed at understanding the different roles of DC subsets in directing effective immune responses to different types of pathogens (Anderson et al., 2018).

## Origins of cross-priming and cross-presentation

By the mid-1960s, lymphocyte function was divided into antibody-dependent humoral immunity and cell-mediated immunity (Cooper et al., 1966). For cell-mediated immunity, an early in vitro assay of lymphocyte function measured the release of C^14^-thymidine from labeled target cells (Vainio et al., 1964), but an improved method based on Cr^51^ labeling soon became universal (Brunner et al., 1968). Zinkernagel and Doherty used this assay to uncover the remarkable finding that in vitro killing of virally infected target cells by CTLs primed in vivo against lymphocytic choriomeningitis was “restricted by the *H-2* gene complex” (Zinkernagel and Doherty, 1974). Their results sparked a series of studies by Bevan that directly led to discovery of cross-priming (Bevan, 1976), later renamed cross-presentation (Carbone and Bevan, 1990).

### Origins from studies of alloimmunization

The process we now call cross-presentation originated from a series of studies examining the in vitro behavior of CTLs induced in vivo against alloantigens, in contrast to the in vivo priming against lymphocytic choriomeningitis infections used by Zinkernagel and Doherty (1974). CTLs induced in vivo by alloantigens were monospecific for recognition of H-2 gene products (Bevan, 1975a). Further, these studies showed that differences in minor histocompatibility (H) alleles (alleles not encoded in the *H-2* locus) could induce CTL responses that were H-2 restricted (Bevan, 1975b). These studies used mouse strains of different backgrounds, such as B10.D2 and BALB/c, harboring different minor H-2 alleles but sharing the same H-2 region. Immunization of BALB/c (H-2^d^) mice with spleen cells from B10.D2 (H-2^d^) mice generated CTLs restricted by H-2^d^, but that were reactive only to antigens derived from the B10 background. This suggested that CTLs recognized antigens “created by an H-2 coded modification of the products of non-*H-2* coded genes—probably minor histocompatibility genes” (Bevan, 1975b) in agreement with the recently discovered *H-2* restriction.

**Figure 2.**
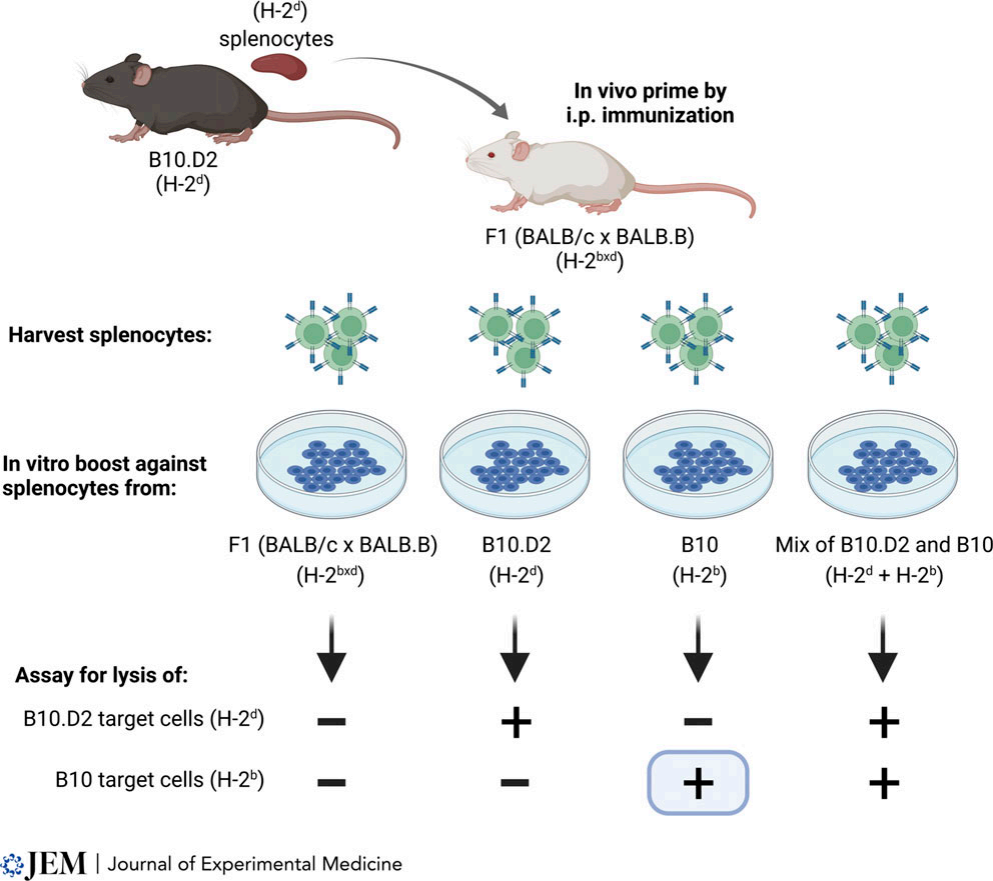
**Cross-priming for a secondary cytotoxic response to minor H antigens.** Splenocytes from B10.D2 mice were used to immunize F1 (BALB/c x BALB.B) mice to induce CTL specific for minor H-2 antigens differing between the B10 and BALB/c backgrounds. After in vivo priming, lymphocytes from immunized F1 mice were boosted in vitro against irradiated splenocytes from F1 (BALB/c x BALB.B) mice, B10.D2 mice, B10 mice, or an equal mixture of splenocytes from B10.D2 and B10 mice. CTL activity was then assayed against target cells from B10.D2 or B10 mice. Cytolysis of B10.D2 targets is consistent with priming by direction of the immunizing B10.D2 cell and does not require an explanation by cross-presentation. In contrast, cytolysis of B10 targets cannot be explained by direct priming by the immunizing cells, suggesting that minor antigens from the B10 background were recognized in vivo by CTLs in the context of the host H-2^b^ allele. This was cross-priming. Adapted from Bevan (1976).

The first experimental evidence for cross-priming arose from somewhat more complicated experiments using F1 mice of mixed H-2 composition (Fig. 2). CTLs were analyzed from F1 (BALB/c x BALB.B; H-2^dxb^) mice that were immunized with cells from B10.D2 (H-2^d^) mice (Bevan, 1976). CTLs primed in this way were able to lyse targets from B10.D2 (H-2^d^) mice, as expected, since these cells were the original immunizing antigen. But somewhat unexpectedly, these CTLs also lysed target cells derived from B10 (H-2^b^) mice, which were syngeneic to the host and of a different H-2 allele from the original immunizing antigen. This cross-priming referred to induction of an H-2^b^–restricted response from immunization with H-2^d^ cells, seemingly inconsistent with H-2 restriction. These results were soon confirmed (Matzinger and Bevan, 1977). One interpretation was that “H-2 restriction does not hold during” the priming stage of CTL induction (Matzinger and Bevan, 1977). However, an alternative interpretation was suggested in 1977 that “a host antigen-presenting cells is involved.” Continuing, Bevan explained that “B10 cells injected into an F1 (C x C.B) are disrupted, and the minor H antigens are picked up by F1 presenting cells which carry both H-2^b^ and H-2^d^.” This process is, essentially, as we describe cross-presentation today. This was a remarkable insight, given that the nature of T cell antigen recognition was still a mystery.

Bevan’s original report of cross-priming cited previous literature that was interpreted as consistent with cross-priming in vivo (Snell et al., 1957; Martinez et al., 1959; Gasser and Silvers, 1972). Snell’s studies of *H* antigens relied on production and analysis of inbred recombinant lines, called congenic resistant lines, tested for susceptibility or resistance to tumors (typically a radiation-induced leukemia) that were derived from other strains. For the majority of congenic resistant lines analyzed, tumor resistance was linked to the *H-2* locus. Non–H-2 loci could also mediate resistance, although this was typically weaker and occasionally allowed tumors to overwhelm the *H* locus barrier. To improve the assay’s discrimination for weaker *H* antigens, Snell developed a new method of typing inbred strains of mice for *H* antigens (Snell et al., 1957). This involved the introduction of a prior immunization of mice with a normal tissue (thymus) from another strain, followed by a challenge with that strain’s leukemia. Use of this method allowed for better discrimination of weaker histocompatibility differences, now called minor H antigens. Cross-priming against minor *H* alleles could explain the basis for this enhanced sensitivity. Notably, Snell himself directed subsequent work confirming in vivo cross-priming, as reported by Murasko (1978). In that study, BALB/c (H-2^d^) mice grafted first with B10 (H-2^b^) tail skin acquired effector cells capable of rejecting a second-set graft of H-2 compatible B10.D2 (H-2^d^) skin. This second-set rejection indicates that CTLs from the BALB/C host recognized minor *H* antigens from the B10 background presented by H-2^d^ alleles, suggesting they were originally induced by BALB/c host APCs through cross-priming.

### Subsequent studies of cross-priming

In the decade following its discovery, cross-presentation was not a topic of intense activity, as greater interest focused on the nature of antigen recognition by T cells. However, as early as 1980, a requirement for antigen processing for CTL responses against minor H-2 was suggested by the inability of glutaraldehyde-fixed allogeneic donor cells to induce robust CTL responses to minor H-2 antigens (Korngold and Sprent, 1980). The subsequent years had rapid progress in the molecular basis for antigen recognition. First, antigens presented by MHC-II molecules were discovered to be inhibited by chloroquine (Ziegler and Unanue, 1982) and shown to be small peptides derived from the antigen (Shimonkevitz et al., 1983; Shimonkevitz et al., 1984; Babbitt et al., 1985). Later, antigens presented by MHC-I molecules were shown also to be small peptides (Townsend et al., 1984; Townsend et al., 1985; Townsend et al., 1986), culminating with the structure of the MHC-I molecule and its peptide ligand (Bjorkman et al., 1987b; Bjorkman et al., 1987a).

Between 1976 and 1990, follow-up studies examined suppression of CTL activity by cross-presented antigens, again suggesting that host APCs “reprocess and present these minor H antigens in conjunction with both H-2 A and H-2 B” (Fink et al., 1983). In 1987, Bevan wrote that a “plausible way to take cellular antigens that are exogenous and to present them as endogenous, class I–associated antigens is via specialized APCs that phagocytose large cellular debris and shuttle the resulting peptide degradation products to their endogenous class I presenting system. Such a phagocytic cell may or may not express class II molecules” (Bevan, 1987).

The clarity of this explanation was not universally appreciated. The prevailing notion that emerged during this time was that MHC-I and MHC-II antigen processing pathways were dedicated solely to either cell-intrinsic or exogenously derived antigens, respectively, with the suggestion that MHC-I “processing may occur in a region of the transitional Golgi specialized for dealing with improperly folded proteins synthesized by the cell” (Germain, 1986). Evidence from in vitro recognition of infected target cells supported an intracellular origin for antigen in loading MHC-I (Morrison et al., 1986). Further, fusogenic activity of a virus was required for its loading onto MHC-I in vitro (Yewdell et al., 1988), which was interpreted as consistent with a purely cell-intrinsic pathway. Importantly, both of these studies were interrogated in only target cells and not professional APCs. By contrast, Bevan reasoned, “if the only cell capable of presenting antigen to class I–restricted T cells is the infected cell itself, then sensitization would have to occur peripherally in the case of a virus that did not productively infect cells in the lymphoid organs” (Bevan, 1987). In short, without cross-priming, CTL responses would need to be primed directly by infected somatic cells, and not by “professional APCs.”

### Revived interest and confirmation

During the early 1990s, interest in cross-presentation was revived as numerous reports confirmed that cross-presentation could occur in vivo under various conditions, while a few studies disagreed. For a third time, Bevan demonstrated in vivo cross-priming, this time using splenocytes pulsed with exogenous proteins as immunogens and conditions similar to his original experiments (Carbone and Bevan, 1990), and also introduced the term cross-presentation for this process. Cross-presentation was soon confirmed by several groups, which showed CTLs can be primed in vivo in response to immunization with protein-pulsed DCs (Inaba et al., 1990) or soluble proteins (Rock et al., 1990), and by proteins delivered using liposomes (Reddy et al., 1992; Collins et al., 1992; Pfeifer et al., 1993). Additionally, cross-presentation was demonstrated to occur in macrophages in vitro (Kovacsovics-Bankowski et al., 1993), although the APC responsible for CTL priming in vivo remained unclear. Some evidence arguing against a need for cross-presentation by professional APCs arose from the demonstration that fibroblasts harboring antigens could induce MHC-restricted CTL responses (Kündig et al., 1995). However, later work would show that CTL responses are not induced directly by virally infected cells and instead rely on bone marrow (BM)–derived cells for this function (Sigal et al., 1999).

The physiologic role of cross-presentation and the identity of the APC responsible for it began to emerge in the next decade. A role for cross-presentation in generating CTL responses to tumors was implied by transference of MHC-I–restricted tumor antigens to BM-derived APCs (Huang et al., 1994). Cross-presentation of exogenous self-antigens was shown to induce deletion of auto-reactive CD8 T cells (Kurts et al., 1997). The capture of apoptotic cells by DCs, but not by macrophages, triggered cross-presentation for the induction of CTL responses in vivo (Albert et al., 1998). Bevan developed a method of delivering antigen in a cell-associated form that is incapable of direct presentation by using β2m^−/−^ cells that were osmotically loaded with OVA as an immunogen (den Haan et al., 2000). With this method, a previously identified DC subset, CD8α^+^ DCs (Crowley et al., 1989; Ardavin and Shortman, 1992; Vremec et al., 1992), but not CD8α^−^ DCs, were shown to be capable of capturing and presenting cell-associated antigens to CD8 T cells in vivo. This was confirmed and extended to soluble protein cross-presentation as well (Pooley et al., 2001). Finally, CD11c-expressing DCs were demonstrated to be sufficient for in vivo cross-presentation in mice engineered to express MHC-I molecules only on DCs (Kurts et al., 2001).

Some evidence has suggested that human pDCs may be capable of cross-presentation in vitro (Hoeffel et al., 2007; Di Pucchio et al., 2008; Segura et al., 2013). Similar evidence was provided for murine pDCs as well (Mouries et al., 2008; Kool et al., 2011). One study showed in vivo priming using antibody-targeting to pDCs with a readout based on activation of transgenic CD4 T cells (Sapoznikov et al., 2007), not relevant to cross-presentation. Another study examined responses to soluble OVA with a readout based on OT-1 CD8 T cells (Shinohara et al., 2006). While these particular experimental settings suggest the capacity for cross-presentation by pDCs, the physiologic relevance to antiviral or antitumor responses was unclear (Colonna and Cella, 2007). Indeed, pDCs were unable to induce endogenous CTL responses unless the antigen was delivered via an anti-SiglecH antibody, despite activation of toll-like receptors (Zhang et al., 2006). Finally, pDCs appear insufficient for autonomous CTL priming, since selective cDC1 lineage ablation abrogates CTL priming to viruses and tumors (Hildner et al., 2008; Durai et al., 2019).

### Cellular and molecular dissection

A period of intense investigation followed these confirmations of cross-presentation (Cruz et al., 2017), largely by analyzing BM-derived DCs (BMDCs) generated in vitro with GM-CSF (Markowicz and Engleman, 1990; Sallusto et al., 1995). Conclusions derived from this system supported both vacuolar and cytosolic pathways involving phagosomes (Houde et al., 2003), phagosome-ER fusion (Guermonprez et al., 2003), and Cathepsin S for generating peptides for a transporter associated with antigen processing–independent vacuolar pathway (Shen et al., 2004). An in vitro role for GAP junctions was suggested for cytosolic delivery of antigens from antigen-bearing cells (Neijssen et al., 2005). Genetic analysis implicated several proteins involved in lysosome and phagosome function, including NOX2 (Savina et al., 2006), Rab27a (Jancic et al., 2007), Rac2 (Savina et al., 2009), IRAP (Segura et al., 2009), Rab3b/c (Zou et al., 2009), Rab34 (Alloatti et al., 2015), and Sec61 (Zehner et al., 2015). In vitro over-expression of the transcription factor EB inhibited cross-presentation by BMDCs (Samie and Cresswell, 2015). However, the involvement of these factors in regulating in vivo cross-presentation remains to be tested (Alloatti et al., 2016; Theisen and Murphy., 2017; Murphy and Murphy, 2022).

Some studies indicated differences in how BMDCs and cDC1 carry out cross-presentation (Briseno et al., 2016; Kretzer et al., 2016). In vivo cross-presentation relies primarily on *Batf3*-dependent cDC1s (Hildner et al., 2008). Roles for in vivo cross-presentation have been shown for the IFN-γ–induced GTPase (Igtp; Bougneres et al., 2009), the vesicular trafficking protein Sec22b (Cebrian et al., 2011), Ras-related GTP-binding protein RAB43 (Kretzer et al., 2016), and the BEACH domain–containing protein WDFY4 (Theisen et al., 2018). DCs from *Igtp*^−/−^ mice lack lipid bodies, structures composed of neutral lipids arising from the ER, and in vivo cross-presentation of cell-associated antigens is reduced about fourfold in *Igtp*^−/−^ mice (Bougneres et al., 2009). Sec22b is a SNARE protein that regulates vesicular trafficking and is highly expressed in cDCs (Cebrian et al., 2011). *Sec22b*^−/−^ mice exhibit reduced in vivo cross-presentation and impaired tumor rejection (Alloatti et al., 2017). However, conditional deletion of Sec22b induced by a CD11c-Cre transgene was reported to not impair in vivo cross-presentation (Wu et al., 2017), but this discrepancy has not yet been resolved. *Rab43*^−/−^ mice showed about a fourfold reduction in cross-presentation in vivo, and the RAB43 protein was localized to cis-Golgi and an unidentified vesicular compartment (Kretzer et al., 2016). WDFY4 was identified in a CRISPR/Cas9 screen for cross-presentation by primary cDC1 generated in vitro by Flt3L treated BM (Theisen et al., 2018). *Wdfy4*^−/−^ mice show normal cDC1 development and normal processing of MHC-II restricted antigens, but exhibited loss of in vivo cross-presentation. This defect in *Wdfy4*^−/−^ mice was accompanied by the inability to make CTL responses against several viruses and tumors and a complete loss of tumor rejection. Notably, BMDCs derived from *Wdfy4*^−/−^ mice showed no defect in cross-presentation in vitro.

## Origins of help for CTL responses

The form of help we now recognize as CD4 T cells licensing cDC1 for CTL priming was very likely the same as the in vitro phenomenon reported by Cantor and Boyse (1975b), despite the use of Ly-1 (CD5) as a marker for the helper cell, rather than CD4 used today. Conducted well before antigen recognition by T cells was understood, this demonstration of T–T interaction may have grown from earlier ideas of T–B cooperation related to the hapten carrier effect discovered at Mill Hill a few years earlier (Mitchell and Miller, 1968). We trace this idea from these origins to its current state.

### Early observations of T–T cooperation

The 1960s saw the identification of a distinct subset of lymphocytes, and the first documentation of the interactions between them. B–T cell cooperation was recognized from the discovery of the hapten-carrier effect in antibody responses (Mitchell and Miller, 1968; Boak et al., 1971). The discovery of help for CTL responses relied on newly available antibodies generated by Boyse against T cell surface proteins Ly-1 (CD5), Ly-2 (CD8α), and Ly-3 (CD8β; Boyse et al., 1968). These antibodies allowed segregation of T cells into functional subclasses (Shiku et al., 1975). Cantor and Asofsky had previously described two populations of cells in graft-versus-host responses in mice that “produced no detectable reactions when injected separately” but were “able to produce significant GVH reactions when combined” (Cantor and Asofsky, 1970). Both populations were later found to be thymus-derived lymphocytes or T cells (Cantor and Asofsky, 1972). The discovery of help for CTL responses was founded on the demonstration that killer activity was mediated by the Ly-23^+^ T cells and that helper activity was exhibited by Ly-1^+^ T cells and that this differentiation occurred prior to antigen encounter (Cantor and Boyse, 1975b). Then, using an in vitro mixed lymphocyte culture, Ly-1^+^ T cells were shown to amplify killer activity of Ly-23^+^ T cells, but without themselves acquiring killer activity (Cantor and Boyse, 1975a). Studies between 1977 and 1983 transformed this original terminology into the CD4 and CD8 classification used today (Chess, 2006).

**Figure 3.**
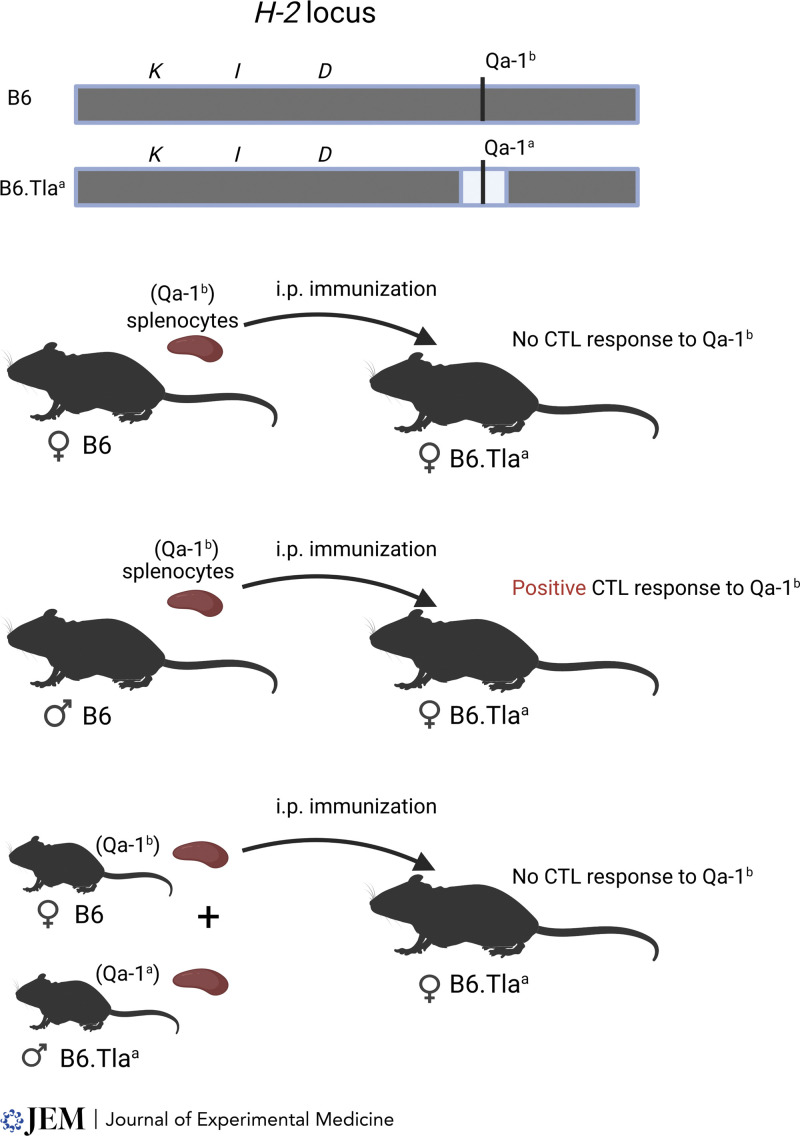
**Linked recognition of helper activity is required for the in vivo generation of cytotoxic T lymphocytes.** A congenic pair of B6 mice were generated differing only at the Qa-1 locus. Original B6 mice express the Qa-1^b^ allele, while the B6.Tla^a^ congenic partner expresses the Qa-1a^a^ allele. Immunization of female B6.Tla^a^ mice with splenocytes from B6 female mice fails to induce a Qa-1^b^ specific CTL response. In contrast, immunization using splenocytes from B-6 male does generate the Qa-1b specific CTL. Male cells carry the additional H-Y antigen that serves as a helper determinant. The requirement for linked recognition was indicated by the inability to generate CTLs using a mixed immunization with B6 female splenocytes and male B6.Tla^a^ splenocytes. This suggested that the H-Y helper determinant and Qa-1^b^ CTL determinant need to be presented on the same cell. Adapted from Keene and Forman (1982).

Other evidence of T–T cooperation was suggested subsequently by several in vivo experiments. These studies used mouse strains with H-2 haplotypes having distinct *I* regions and K/D regions of known permissiveness for CTL responses to vaccinia virus (Zinkernagel et al., 1978), male H-Y antigen (von Boehmer et al., 1978, or the Qa-1 alloantigen (Keene and Forman, 1982). Each study argued that strong CTL responses required a combination of permissive K or D allele with a permissive I region, which was interpreted as supporting a model of helper and CTL cooperation. Although these studies were not without some degree of ambiguity, Keene and Foreman suggested explicitly that the helper and CTL determinants should be expressed on the same cell (Keene and Forman, 1982), supporting linked recognition as a basis for help in CTL responses (Fig. 3).

### A three-cell-type model of linked recognition

Subsequent work by Mitchison and O’Malley provided evidence for a three-cell-type model of linked recognition, involving a pre-CTL, helper T cell (T_H_ cell), and APC (Mitchison and O’Malley, 1987). In elegant fashion, adoptive transfers of alloreactive CTLs or helpers of different H-2 specificity were used in concert with appropriate H-2 recombinant mice to show that direct T–T cell cognate interactions were not required for help. The authors concluded that “an APC copresenting two epitopes may create a microenvironment that succeeds in bringing together two types of T cells.” Independent confirmation was provided when allogeneic responses to the class I H antigen Qa1 and the minor H antigen H-Y were also found to require help, without which a tolerant state was acquired (Guerder and Matzinger, 1992). However, how such a “microenvironment” would mediate help was unclear. For example, help might arise from co-localization of helper and killer precursors, allowing for efficient delivery of helper-derived cytokines, such as IL-2 to CTLs (Fearon et al., 1990). Alternately, at this time it was recognized that T cells can receive co-stimulatory signals from APCs (Lafferty and Cunningham, 1975). Thus, T helpers may activate APCs (Beller and Unanue, 1980; Walker et al., 1982) as a means of help. On this latter foundation, Matzinger proposed the alternate mechanism that help is “routed” through and by the APC, rather than being delivered directly from helper to CTL (Guerder and Matzinger, 1992).

### CD40 stimulation activates DCs

CD40 was identified in 1986 through antibodies that induced B cell proliferation (Clark and Ledbetter, 1986), but was soon found to be expressed on human tonsillar DCs as well (Hart and McKenzie, 1988). CD40 stimulation was later shown to increase expression of MHC-I and -II molecules and costimulatory molecules CD80 and CD86 on DCs (Sallusto and Lanzavecchia, 1994; Caux et al., 1994). Importantly, Cella showed that CD40 stimulation of DCs enhanced their capacity to induce T cell proliferation and cytokine production (Cella et al., 1996). CD40 ligand (CD40L), identified by expression cloning (Armitage et al., 1992), was found to be expressed on activated T_H_ cells (Noelle et al., 1992; Lederman et al., 1992). In this period, the CD40–CD40L signaling axis was actively studied in the context of B–T cell cooperation (Armitage et al., 1992).

Three simultaneous reports provided evidence that CD4 T cell help for CTL responses in mice was mediated by stimulating CD40 signaling in an APC (Schoenberger et al., 1998; Ridge et al., 1998; Bennett et al., 1998). The approaches used included the depletion of CD4 T cells to abrogate help, provision of CD40 signaling using anti-CD40 antibodies, and analysis of CTL responses in mice with germline deficiencies in CD40 and CD40L. Together, these studies supported the three-cell-type model with the addition that CD40 signaling delivered help to CTLs by activating CD40 signaling in an APC. Reliance on germline deficiencies precluded precise identification of the cellular site of CD40 signaling, although B cells were explicitly excluded (Schoenberger et al., 1998). Another study implicated DCs as the target of anti-CD40 stimulation as a substitute for help, but the DCs used in this setting were uncharacterized (Ridge et al., 1998). Heath and colleagues suggested that the target of CD40 signaling was the “cross-priming APC,” but direct evidence for its identity was not possible at that time (Bennett et al., 1998).

In contrast, another study argued against the APC as the target of CD40 signaling. In examining CTL responses to H-Y antigen, Tanchot and colleagues found that CD40 signaling was important for memory, but not the primary CTL response (Bourgeois et al., 2002). Moreover, while providing evidence for a three-cell-type model, this study argued that CD40 signaling acted directly within CTL, but not the APC. The experimental basis for this claim relied on adoptive transfers of H-Y–bearing APC from WT or CD40^−/−^ mice, introduced into recipient mice harboring CD4 and TCR-transgenic H-Y specific CD8 T cells. No difference between responses was seen in mice receiving APCs from WT or CD40-deficient APCs, a negative result interpreted as excluding a requirement for CD40 expression by APCs in mediating help. However, as discussed above, H-Y antigens from CD40^−/−^ APCs can still be cross-presented by the CD40-sufficient host APCs. Nonetheless, subsequent studies using infectious model systems argue against these results, supporting the requirement for CD40 signaling in the APC as the primary mechanism for help in CD8 T cell memory (Lee et al., 2003; Sun and Bevan, 2004). However, the structure of these studies did not directly test the effect of selective CD40 deficiency on CD8 T cells. For example, CD40-deficient CD8 T cells were only tested in *Listeria* infection in a setting that was independent of CD40-mediated help (Sun and Bevan, 2004).

### Identification of the APC mediating CD4 T cell help

In vitro analysis (Smith et al., 2004) and intravital imaging (Eickhoff et al., 2015; Hor et al., 2015) suggested that the relevant target of CD4 help for CTL responses is the cDC1 subset, which is uniquely responsible for priming CD8 T cells to tumors (Hildner et al., 2008; Theisen et al., 2018), but did not demonstrate the involvement of CD40 signaling in vivo. Direct in vivo evidence for the requirement of CD40 expression on cDC1 for help-dependent rejection of tumors was recently obtained using a cDC1-specific Cre deleter mouse strain, *Xcr1*^Cre^, crossed to *CD40*^fl/fl^ or *MHC-II*^fl/fl^ mouse strains (Ferris et al., 2020). Mice with cDC1-specific inactivation of CD40 failed to reject tumors normally rejected by WT mice, in a system where tumor rejection requires both CD8 priming and CD4 help (Ferris et al., 2020). In the same system, cDC1-specific inactivation of MHC-II expression severely reduced the expansion of endogenous tumor-specific CD8 T cells and impaired tumor rejection. These results support a role for CD40 signaling activated by CD4 T cells in cDC1 to provide help for CD8 T cells during tumor challenge.

One target of CD40 signaling proposed as providing help for CTL responses involves the CD70/CD27 signaling axis (Taraban et al., 2004; French et al., 2007; Feau et al., 2012; Ahrends et al., 2017; Borst et al., 2018). Several studies have used CD70 blockade by antibody (French et al., 2007; Feau et al., 2012; Keller et al., 2008; Ahrends et al., 2016) or examined CD70 or CD27 germline deficiencies (Munitic et al., 2013; Hendriks et al., 2000) to demonstrate a role for CD70 expression on cDC1 for enhancing CTL responses. However, these studies could not pinpoint the cellular action of CD40 and CD70. Toward this goal, another study examined *Batf3*^−/−^ *Cd70*^−/−^ mixed BM chimeras to test the role of CD70 on cDC1 (Oba et al., 2020), but did not examine responses of endogenous T cells to tumors. No study has yet tested the specific requirement for cDC1-specific CD70 expression using a conditional deletion system in vivo. Thus, it remains unclear whether induction of CD70 on cDC1 is fully responsible for mediating CD40-dependent help for CTL responses.

## The road ahead in DCs, cross-presentation, and help

Current work in DCs is very broad and comprises open questions related to subset heterogeneity, functional specialization, and developmental pathways. Other questions related to alterations of DC in response to pathologic processes and the degree of individual variability within the human population. Within the area of functional specialization, the mechanism by which different DC subsets support alternative T helper cell responses remains undefined. For example, protection against *Toxoplamsa gondii* in mice requires the cDC1 subset presumably based on its superior IL-12 production (Mashayekhi et al., 2011). However, the molecular basis for this remains unclear (Kim et al., 2020). Similarly, cDC2 appears specialized for driving T_H_17 (Satpathy et al., 2013) and T_H_2 cell responses (Kumamoto et al., 2013; Gao et al., 2013; Williams et al., 2013) against various pathogens, but again the underlying mechanisms are unknown. Alternative explanations include those relying on cytokine bias, for example, with cDC2 acting as neutral agents in early CD4 T cell priming, thereby avoiding an early commitment toward a T_H_1 cell outcome. However, other explanations could involve differential localization within lymphoid tissues or differences in antigen processing. This field is in its early stages since reagents for complete cDC2-specific lineage ablation or gene inactivation are lacking.

Much of the literature on cross-presentation relies on analysis of BMDCs developed in vitro using GM-CSF, but the physiologic relevance of this system has recently been challenged (Helft et al., 2015). Many gene candidates for participation in cross-presentation that derive from these studies have yet to be tested for their impact on in vivo cross-presentation. The cDC1 lineage appears primarily responsible for in vivo cross-presentation, but analysis of its cellular pathway for cross-presentation is incompletely defined. Unique surface receptors expressed by cDC1, such as CLEC9A, may contribute to its capture and processing cell-associated antigen (Sancho et al., 2009), but these do not appear to be autonomously sufficient for this activity. The intracellular protein WDFY4 is a stringent requirement for in vivo cross-presentation. However, WDFY4 is expressed by cDC1 and cDC2, and again does not explain cDC1-specific cross-presentation. WDFY4 itself remains an enigma, with virtually nothing known regarding its cellular function. Future work will be required to identify the molecular pathways that connect receptors such as CLEC9A to the intracellular trafficking pathways within cDC1 that deliver their cargo to MHC-I loading compartments.

While CD40 is clearly a control hub in mediating CD4 help for CTL responses, the underlying cellular mechanism in cDC1 remains obscure. For example, the target genes induced by CD40 signaling in cDC1 remain largely unidentified, and which of these targets contributes to CTL responses remains incompletely defined. The induction of any one gene, such as *Cd70*, may not fully explain the complete effect of cDC1 licensing on CTL responses. Further, while CD40 signaling in cDC1 appears important for help, other factors may include the induction of cytokines and chemokines by cDC1 or CD4 T cells (Mackey et al., 1998; Castellino et al., 2006), or the amplification of other signals in cDC1 (Greyer et al., 2016; Schulz et al., 2000), or the enhanced survival or accumulation of these cells (Bjorck et al., 1997; Miga et al., 2001). Also, CD4 T cells may not be the exclusive cells responsible for licensing cDC1. Indeed, deletion of MHC-II on cDC1, which prevents cognate interactions with CD4 T cells, had less impact on CTL priming than did the deletion of CD40 on cDC1 (Ferris et al., 2020). This result may suggest that alternative cells, such as NK T cells that react with lipids presented by CD1 molecules, may also license cDC1 (Fujii et al., 2002). Alternately, CD4 T cells may function in a non-cognate manner to provide generalized cDC1 licensing in some settings (Pasqual et al., 2018).

In the 1970s, the connection between the discovery of DCs, cross-priming, and help for CTL responses was not fully appreciated, but these are now recognized as being connected elements embodied in the three-cell-type model (Mitchison and O’Malley, 1987). Decades of subsequent investigation have left this model conceptually intact, but it is now being seen with increasing resolution of detail. Nonetheless, the picture is not complete, and important aspects of this model are in need of further refinement.
